# Chronic Obstructive Pulmonary Disease (COPD) During the Two Last Years of Life – A Retrospective Study of Decedents

**DOI:** 10.1371/journal.pone.0084110

**Published:** 2013-12-19

**Authors:** Britt-Marie Sundblad, Sven-Arne Jansson, Lennarth Nyström, Peter Arvidsson, Bo Lundbäck, Kjell Larsson

**Affiliations:** 1 Lung and Allergy Research, Institute of Environmental Medicine, Karolinska Institutet, Stockholm, Sweden; 2 Krefting Research Centre, Institute of Medicine, Sahlgrenska Academy, University of Gothenburg, Gothenburg, Sweden; 3 Department of Public Health and Clinical Medicine, Umeå University, Umeå, Sweden; 4 Astma/COPD, GlaxoSmithKline Sweden, Stockholm, Sweden; Clinica Universidad de Navarra, Spain

## Abstract

**Background:**

Little is known about the management of patients suffering from chronic obstructive pulmonary disease (COPD) during the last years of life. The aim of the study was to describe how management of COPD is performed in Sweden during the last two years of life.

**Methods:**

From the nationwide Cause of Death register all individuals with COPD as the underlying cause of death during two years were identified in one sparsely and one densely populated area of Sweden. Data were collected from medical records using a pre-defined protocol, especially developed for this purpose.

**Results:**

Of 822 individuals with COPD as underlying cause of death, medical records from 729 were available. The COPD diagnosis was based on lung function measurements in approximately half of the patients and median age at COPD diagnosis was 74 years (range 34-95). Women died at younger age, median 78 years (range 52-96) than did men (80 years (51-99)). The median survival time from diagnosis to death was 6 years in men and women in both areas. Among women and men 8.3% and 4.3% were never smokers, respectively. The structure of COPD management differed between the two areas, with utilization of physiotherapists, dieticians and working therapists being more used in the northern area, likely because of differences in accessibility to care institutions.

**Conclusions:**

In Sweden COPD is mostly diagnosed late in life and often not verified by lung function measurements. Opposite to the general population, women with COPD die at a lower age than men.

## Introduction

Chronic obstructive pulmonary disease (COPD) is rare before the age of 40 and becomes increasingly prevalent with increasing age. From a global perspective the prevalence of COPD has been estimated to approximately 14% in the population above the age of 65 years [1] and is estimated to be the third most common cause of death in 2020 [2]. In Sweden more than half a million persons have COPD [3] and close to 3,000 die every year as a consequence of the disease.

The COPD diagnosis requires measurement of lung function. However, the diagnosis is often missed due to the reluctance to perform lung function tests in risk groups, and under-diagnosis is common worldwide. In Sweden only one out of five persons with chronic airflow limitation had physician-diagnosed COPD [4,5,6]. Under-diagnosis of COPD is likely also caused by other co-morbid conditions, primarily cardiovascular diseases, implying treatment of the co-morbidity without noticing COPD [7].

If COPD is not diagnosed at an early stage preventive measures like smoking cessation and treatment will be delayed. Early smoking cessation is of utmost importance for future disease progression and COPD exacerbations are associated with rapid lung function decline and increased mortality [8]. Early onset of treatment of exacerbations therefore improves the prognosis in COPD [9,10]. There are studies indicating that pharmacological treatment with the combination of an inhaled corticosteroid and a long-acting β_2_-agonist [11] and treatment with tiotropium [12] may reduce mortality in COPD. 

The knowledge about the circumstances prior to death is sparse and there is few data on how COPD patients are treated during final stage of life. The aim of this study was to describe the management of COPD patients during their last two years, based on information obtained from patient medical records in two large Swedish areas covering 19% of the Swedish population. A further aim was to study gender and regional differences with regard to the two last years of life in patients with COPD.

## Materials and Methods

### Study design

A retrospective study of all patients with COPD (ICD10: J43-J44) as underlying cause of death during two years in two large areas of Sweden was conducted. The National Board of Health and Welfare, Center of Epidemiology, Sweden, gave ethical approval and allowed examination of the medical records data (protocol 34-4451/2007). As the medical records relate to deceased subjects the regional ethics committee judged that ethical considerations were not to be conducted by the committee (regional ethics committee in Stockholm 2008/1:3, protocol 2008/311.31). The committee was then asked to give an advisory remark in which it stated that there are no ethical obstacles related to the study. Informed consent (for medical records examination) was obtained from the senior consultant in charge of each medical service area.

### Study area

**Figure 1:**
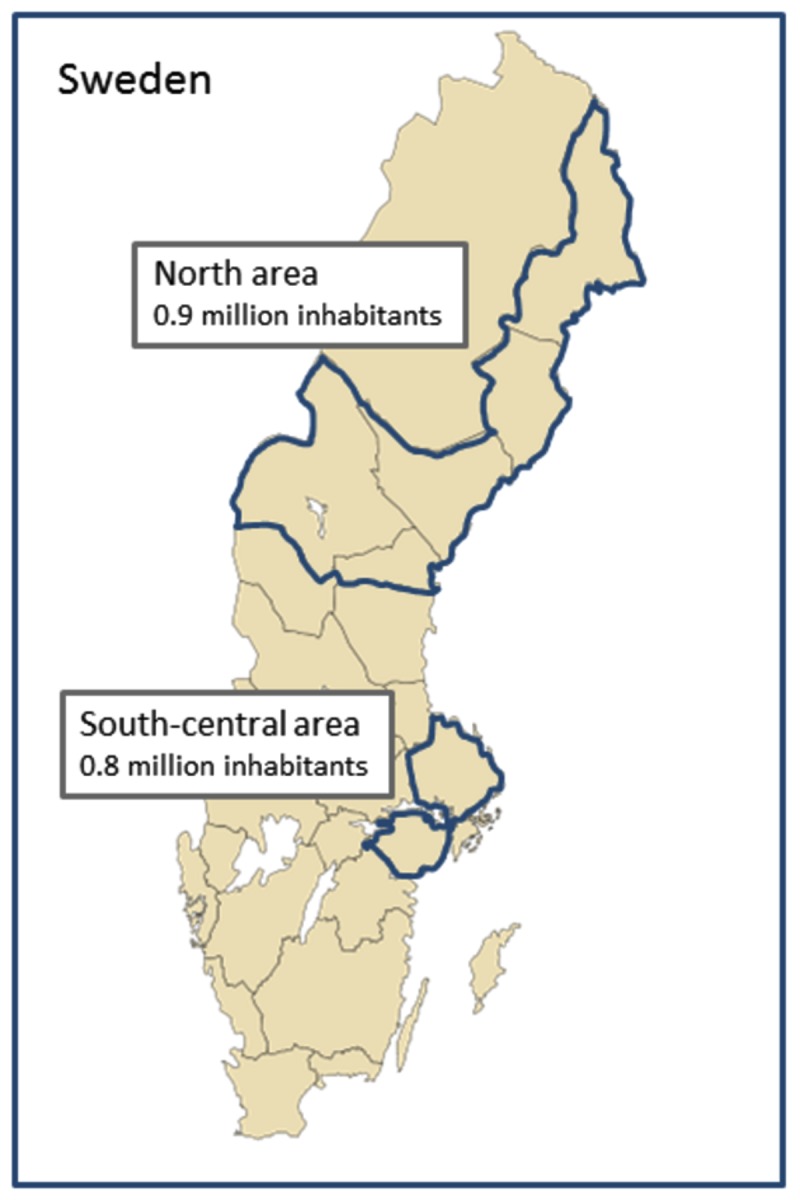
Map of Sweden with the two study areas.

The study covers both south-central and north areas of Sweden (Figure 1). The rural area includes the four northernmost counties Norrbotten, Västerbotten, Västernorrland and Jämtland. This area covers more than 50 % of the area of Sweden but is sparsely populated with a population of 900,000 with 80 % living in towns with 2,000-100,000 inhabitants. The south-central area includes two counties, Uppsala county and Södermanland, and three municipalities (Botkyrka, Huddinge and Södertälje) outside, but close to the capital Stockholm. This area had a population of 800,000 with more than 90 % living in urban areas. At the time of study, 19 % of the Swedish population lived in the study areas.

### Data collection

The deceased cases were retrieved from the nationwide Cause of Death register containing information on civic number and name, date of death, underlying and contributory cause of death and bases for classification of cause of death. In Sweden each county has a computerized system for the medical record for in- and outpatient care using the unique civic number as identity from which information for the two years preceding death was retrieved in each county.

Data were collected from the patient records of all patients who had died due to COPD in 2003 - 2004. One person carried out the data collection in the northern area and two persons in the south-central area. A pilot study was performed in one county in Northern Sweden and two municipalities in the south-central in order to test whether the method of data extraction was well adapted to its purpose. 

### Measure instrument

A pre-defined protocol was developed for collection of information regarding COPD from the medical records and included information on date of diagnosis and spirometry at diagnosis, concomitant diagnoses during the two years preceding death and at the time of death. Furthermore, information about place of death, smoking habits, diagnostic activities and monitoring of the disease were included. Information from chest X-ray, chest CT-scan, electrocardiography (ECG) and echocardiography was also collected.

### Statistical methods

Data are presented as median (range) or mean (standard deviation, SD). To test whether there was an association between place of death and smoking habits and sex and study area chi-square test was used. Differences in the prevalence between groups were analysed using Student’s t-test and differences in the median between groups were analysed using Mann-Whitney U test. A p-value < 0.05 was considered statistically significant. Statistical comparisons were assessed by STATISTICA (StatSoft® Scandinavia AB, Uppsala, Sweden). 

## Results

In the study areas 822 patients died with COPD as the underlying cause of death during the study period. Sufficient information in the medical records was found for 729 (89%) of the patients (table 1). 

**Table 1:** Number of inhabitants December 31, 2002, number of deceased with COPD as the underlying cause of death 2003-2004 and number (%) of deceased 2003-2004 with medical records with sufficient information by county.

County	Number of inhabitants 2002-12-31	Number of deceased 2003-2004	Medical records with sufficient information		
			Number	%	% women
*North area*					
Norrbotten	253 632	173	167	97	48.5
Västerbotten	255 230	97	93	96	43.0
Västernorrland	244 319	128	111	87	56.8
Jämtland	127 947	77	70	90	41.4
*South-central area*					
Stockholm	241 286	79	75	95	54.7
*Huddinge*	86 457				
*Botkyrka*	79 613				
*Södertälje*	75 216				
Södermanland	259 006	155	106	68	47.2
Uppsala	298 655	113	107	95	45.8
Total	1 680 075	822	729	89	48.4

**Figure 2:**
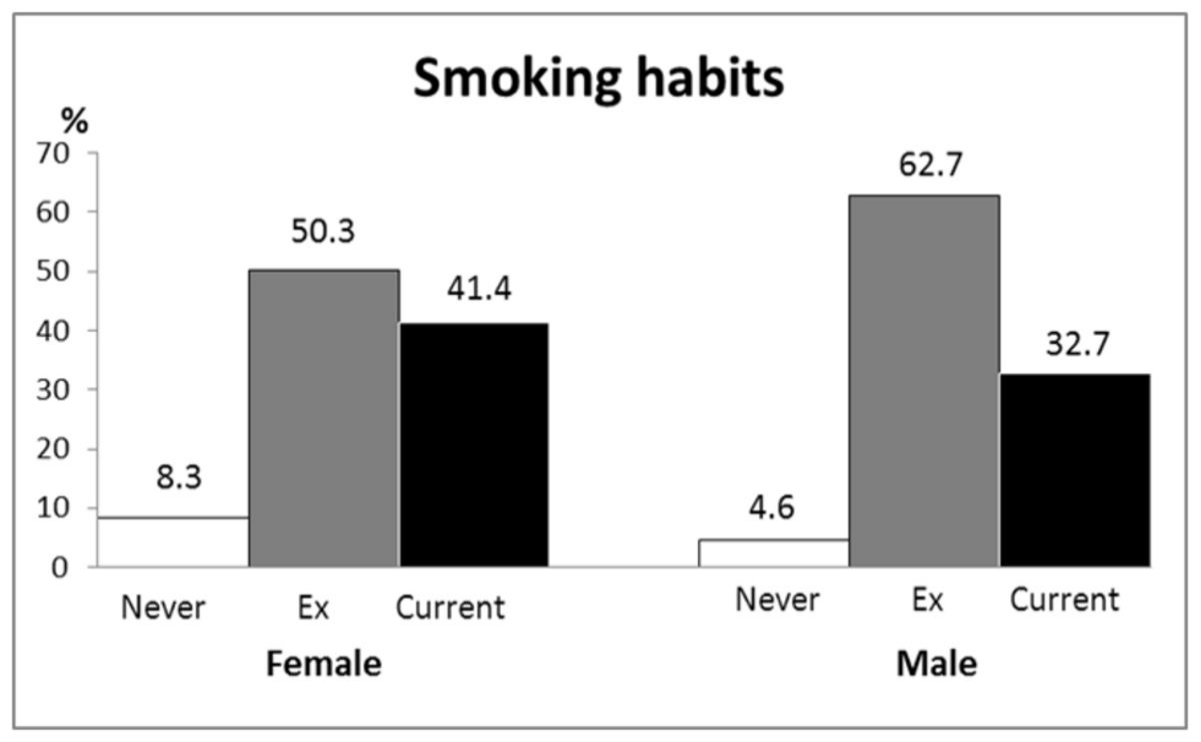
Smoking habits during the last two years before death in 325 women and 350 men with COPD. There is a gender difference in never-smokers (p=0.04), ex-smokers (p=0.001) and current smokers (p=0.02).

Median age at death was 79 years (range: 51-99); 78 years (52 - 96) in women and 80 years (51 - 99) in men (p=0.02) and similar in the two areas (p=0.10; table 2). The median age when receiving the COPD diagnosis was 74 years (34-95); 72 years (34-95) in women and 75 years (41-95) in men (p=0.004). Two thirds (68%) died at hospital, 18% at nursing homes and 13% at home and there was no difference between women and men. Duration from diagnosis to death was similar in men and women and in the two areas (table 2). Eight patients received their diagnosis post mortem. Information about smoking habits at the time of death was found in 675 (93%) patients. The prevalence of current smokers at the time of death was 41 % in women and 33 % in men (p=0.02; table 2, Figure 2,). Of the patients who died due to COPD 8.3% of the women and 4.6 % of the men were never-smokers (p=0.04).

**Table 2:** Patient characteristics.

		Sex			Area		
	All (n=729)	Women (n=353)	Men (n=376)	P-value^1^	North (n=441)	South-central (n=288)	P-value^2^
Age at death, years, median	79.3	78.3	80.4	0.017	78.7	79.9	0.10
(range)	(51-99)	(52-96)	(51-99)		(53-99)	(51-96)	
*Place of death (n=501)*							
At home, %	13.0	14.4	11.7	0.47	13.5	12.0	0.64
Local nursing home, %	17.6	16.5	18.5	0.54	17.3	18.0	0.82
Hospital, %	67.8	67.8	67.9	0.91	67.0	69.4	0.49
Other place, %	1.6	1.3	1.9	0.54	2.2	0.6	0.16
*Smoking prevalence (n=675)*							
Current smoker, %	36.9	41.4	32.7	0.019	38.1	35.0	0.51
Ex-smoker, %	56.7	50.3	62.7	0.001	54.5	60.1	0.13
Never-smoker, %	6.4	8.3	4.6	0.044	7.4	4.9	0.13
Pack years, median	45.0	35.3	50.0	<0.001	41.3	49.0	0.27
(range)	(3.1-140)	(3.1-140)	(3.9-126)		(3.9-140)	(3.1-126)	
*Diagnosis (n=729)*							
COPD verified by spirometry, %	47.5	48.2	46.8	0.42	58.0	31.3	<0.001
COPD stated in medical records, %	88.3	91.5	85.4	0.37	87.5	95.1	0.007
Emphysema stated in medical records, %	9.6	9.2	10.1	0.55	7.5	12.8	0.016
Time from diagnosis to death, years, median (n=660)	6.0	6.1	5.9	0.95	6.2	5.6	0.33
(range)	(0-36)	(0-29)	(0-36)		(0-36)	(0-23)	
Age at COPD diagnosis, years, median (n=660)	73.7	71.7	74.7	0.039	72.5	74.8	0.004
(range)	(34-95)	(34-95)	(41-95)		(34-95)	(45-95)	

Results are given as median (range) or as percent. P-value for difference by 1.sex and 2.area.

The time of the first spirometry, and thereby the time of COPD diagnosis could be determined in 346 (47%) patients, 58% in the north and 31% in the south-central area (table 2). Lung function measurements during the two last years of life were available in 175 (24%) patients in whom spirometry had been performed 1 - 13 times. Data on diffusion capacity measurement was found in only 13 patients. 

During the two last years of life, oxygen saturation assessed by pulsoxymetry was registered in 576 (79 %) patients with a median number of 9 measurements (1 - 245; table 3) and blood gas analyses were performed 4 times (1 - 106) in totally 505 (69 %) patients. Chest X-ray examination was performed 4 times (1 - 19) in 495 (68%) and chest CT-scan once (1 - 4) in totally 100 (14%) patients. The most common outcomes of chest X-ray examinations were COPD (61%), not specified noduli (55 %) and heart failure (49 %), and the most common outcome of CT-scans were emphysema (57 %), not specified noduli (30 %), parenchymal changes (28 %) and pleura plaque (23 %).

**Table 3:** Lung function and oxygen saturation measurements at diagnosis and during the two last years of life.

		Sex		Area	
	All (n=729)	Women (n=353)	Men (n=376)	North (n=441)	South-central (n=288)
*Lung function, first in records (at COPD diagnose*)					
FEV_1,_ L	1.13 (0.63)	0.85 (0.51)	1.31 (0.69)	1.14 (0.63)	1.10 (0.65)
number	346	170	176	256	90
VC or FVC, L	2.28 (0.98)	1.85 (0.73)	2.72 (0.98)	2.31 (0.96)	2.18 (1.04)
number	322	158	164	250	72
FEV_1_/ VC or FVC	0.51 (0.16)	0.51 (0.17)	0.49 (0.16)	0.50 (0.16)	0.52 (0.17)
number	320	154	166	248	72
*Lung function, last before death*					
FEV_1,_ L	0.94 (0.65)	0.80 (0.52)	1.08 (0.72)	0.95 (0.65)	0.93 (0.64)
number	175	86	89	103	72
VC or FVC, L	1.99 (1.04)	1.63 (0.89)	2.31 (1.06)	1.95 (0.83)	2.05 (1.33)
number	152	72	80	96	56
FEV_1_/ VC or FVC	0.49 (0.19)	0.49 (0.17)	0.48 (0.21)	0.49 (0.20)	0.49 (0.18)
number	151	69	82	96	55
*Oxygen saturation*					
SaO_2_, %, First measurement	88 (10)	87 (12)	89 (8)	88 (11)	88 (10)
number	576	277	299	385	191
SaO_2_, %, Last before death	86 (12)	85 (13)	86 (11)	85 (12)	87 (11)
number	570	274	296	383	187

Results are given as mean values (standard deviation, SD).

Information about acute COPD exacerbations during the last two years of life was found in 83% of the patients in the northern, more sparsely populated area and 32% in the south-central area (p<0.001;table 4). The median number of exacerbations was 3 (1 - 26) during this period of two years.

**Table 4:** Contacts with representatives of the COPD-team, exacerbations, co-morbidities and smoking cessation activities.

		Sex			Area			
	All (n=729)	Women (n=353)	Men (n=376)	P-value^1^	North (n=441)	South-central (n=288)	P-value^2^
*COPD care*							
Visit to a nurse, %	29.6	30.9	28.4	0.47	39.2	14.9	0.67
median (range)	3 (1-69)	2 (1-69)	3 (1-57)		3 (1-69)	3 (1-7)	
Visit to a physiotherapist, %	29.5	29.2	29.8	0.21	34.2	22.2	<0.001
median (range)	2 (1-53)	2 (1-53)	2 (1-21)		1 (1-18)	4 (1-53)	
Visit to others, % (e.g. psychologist)	36.4	36.5	36.2	0.59	43.5	25.3	<0.001
median (range)	3 (1-37)	3 (1-22)	3(1-37)		2 (1-37)	6 (1-37)	
Acute visit to a doctor, %	60.9	58.4	63.3	0.78	50.3	77.1	<0.001
median (range)	2 (1-28)	2 (1-28)	2 (1-17)		2 (1-12)	2 (1-28)	
Planned visit to a doctor, %	42.8	43.1	42.6	0.37	54.4	25.0	0.055
median (range)	2 (1-18)	2 (1-18)	2 (1-17)		2 (1-18)	2 (1-17)	
*Exacerbations*							
Exacerbations, %	62.7	63.7	61.2	0.39	83.0	31.6	<0.001
median (range)	3 (1-26)	2 (1-25)	3 (1-26)		3 (1-26)	1 (1-12)	
Hospitalization days due to COPD	23	24	22	0.77	27	17	0.72
median (range)	(1-694)	(1-694)	(1-679)		(1-694)	(1-512)	
*Co-morbidity*							
Hospitalization due to cardiovascular disease, %	24.7	24.1	25.3	0.81	24.5	25.0	0.47
median (range)	1 (1-12)	1 (1-4)	1 (1-12)		1 (1-4)	1 (1-12)	
Hospitalization, other co-morbidity, %	35.7	37.4	34.0	0.60	40.1	28.8	0.16
median (range)	2 (1-4)	2 (1-4)	2 (1-4)		1 (1-4)	2 (1-4)	
Information about smoking cessation activities in medical records	380	184	196	0.94	306	74	<0.001

P-value for test of difference by 1.sex and 2.area.

Electrocardiography (ECG) was registered in medical records in 473 (65%) patients, median 3 times (1-24) with arrhythmia (96%) and increased left ventricular load (67%) as the most common interpretations. Echocardiography was registered in 120 (16%) patients, median 1 time (1-6) and hypertrophy/insufficiency was found in 57%, valve disorders in 54% and pulmonary hypertension in 48% of the patients.

The number of visits to different care providers including physiotherapists, dieticians and working therapists was higher in the northern area compared to the south-central area (p<0.001) with one exception, the number of acute visits to a doctor was more frequent in the south-central area (p<0.001; table 4).

## Discussion

In the present study, based on a sample representing 19 % of the Swedish population, we found that the median age at death in patients dying due to COPD as the direct cause was 79 years, 78 years in women and 80 years in men. This result was unexpected as the mean lifetime for Swedish women was 83 years and for men 78 years at the time of the study. In contrast to the common situation, the survival was much shorter in women than in men. In a prospective study by de Torre et al a shorter survival was found in men than in women with mainly mild and moderate COPD [13]. In that study approximately half of the patients died due to respiratory causes and adjustment for the difference in age did not alter the conclusions. These data are supported by a Canadian observational study in which survival was increased in women compared with men [14]. Analysis of data from the TORCH-study showed no difference in the risk of dying between women and men with COPD [15]. In the present study it is likely that most of the patients had severe or very severe disease as COPD was the direct cause of death in all cases. 

In agreement with the present data it has been claimed that women are more susceptible to tobacco smoke than are men [16]. Lung function decline over time is faster in female smokers and ex-smokers than in male smokers and ex-smokers [17] and female smokers seem to develop COPD earlier than smoking men [18]. Further, lung growth during adolescence is likely more retarded in female than in male smokers [19]. Our finding that COPD diagnosis was established earlier in life and that death from the disease occurred at a younger age in women than in men, despite a lower cumulative exposure to tobacco smoke, indicates that women may be more sensitive to tobacco smoke than men. The risk for cardiovascular co-morbidity and the risk for hospitalization have also been found to be higher in women than in men with COPD [20,21]. In our study there was, however, no significant gender difference in hospitalization due to either cardiovascular or other co-morbidities during the two last years of life. Data on gender mortality differences in COPD patients treated with long term oxygen indicate that women survive longer than men [22,23], results which are contradicted by others [24]. In the present study we have no information about long term oxygen treatment but there was no gender difference in patients, who occasionally had been treated with oxygen during their last two years in life.

The high age at death from COPD may appear somewhat surprising. The low awareness of COPD in primary care contributes to a delay of the diagnosis, which certainly leads to a substantial number of missed diagnoses. The COPD diagnosis was established at the age of 74 years. At the time of the study approximately one fifth of the subjects with COPD in Sweden have received a diagnosis by a physician [4,5,6]. It is therefore reasonable to assume that there are a substantial number of subjects who have COPD and even die from the disease without being diagnosed. In a recent prospective Swedish population study the mortality over a three-years period was approximately doubled among subjects with COPD compared with those who did not have COPD [10]. From our results we can thus conclude that the information obtained from the nationwide Cause of Death register does not give the full picture with regard to COPD mortality. It seems obvious that there are a number of individuals who suffer from COPD and die from it without ever getting the diagnosis.

At the time of death more than 40 % of the women and one third of the men were still smokers. This reflects the general trend in smoking habits in Sweden where smoking has become more common in women than in men. The higher smoking prevalence in women in our study may also contribute to the finding of lower age at death in women.

Although the COPD diagnosis must be based on lung function tests we were able to find data on lung function on less than half of the patients. It could, however, not be excluded that the diagnosis was based on spirometry in a greater proportion of the patients. Thus, lung function tests may have been conducted although we were not able to find the results or the results of lung function tests were not registered in the medical records. Even if this adds to the number of patients in whom spirometry have been performed, we conclude that COPD is often diagnosed without lung function measurement. 

Furthermore, in only half of the patients in whom spirometry had been performed for diagnosis repeated lung function measurements were conducted during the two last years of life. This clearly indicates that lung function tests are not included in the standard procedures in the diagnosis and follow up of patients with COPD in Sweden. It was also demonstrated that spirometry was much less often performed in the south-central area than in the northern area. As every county in Sweden makes their own decisions about how to organize medical care, development of practice differs between counties implying inequalities in disease management.

In this sample of patients who died due to COPD, 6.4 % had never been smokers and among them two thirds were women. In the present study we have no information of other risk factors for COPD, such as occupational or other exposures including environmental tobacco smoke. It is, however, intriguing that the mortality due to COPD was twice as high in never-smoking women compared to never-smoking men. This finding is in line with a recent Swedish publication showing that COPD is more common in women than in men who never smoked [25]. The reason for this is unknown. Considering smoking habits in Sweden in the 50s and 60s, it could be anticipated that a substantial proportion of never-smoking women, who died from COPD in the beginning of the 2000s, have been exposed to environmental tobacco smoke.

The number of acute visits to a doctor was higher in the south-central area than in the north. The reason for this is most likely a consequence of a greater availability to care providers in the south-central area. In the northern part of Sweden the distance to a doctor is often much longer. However, the utilization of other care providers such as physiotherapists, dietician and working therapists seemed to be higher in the north. This finding possibly indicates a more urgent need for building COPD teams in more sparsely build up areas with longer distances between health care givers. 

In conclusion, in decedents with COPD as the direct cause of death COPD had been diagnosed late in life and the diagnosis was not verified by spirometry in a half of the patients. Smoking status, oxygen saturation and acute exacerbations were monitored in a majority of the patients during the two last years of life. The mean survival time from diagnosis to death was six years among women and men but women were diagnosed and died at a younger age, the latter being opposite to the situation in the general population. 
